# Anilinium 3,4-dihy­droxy­benzoate

**DOI:** 10.1107/S1600536812032758

**Published:** 2012-07-28

**Authors:** Si-Ming Zhu

**Affiliations:** a School of Light Industry and Food Science, South China University of Technology, Guangzhou 510641, People’s Republic of China

## Abstract

The asymmetric unit of the title salt, C_6_H_8_N^+^·C_7_H_5_O_4_^−^, contains two anilinium cations and two 3,4-dihy­droxy­benzoate anions. An intra­moleculer O—H⋯O hydrogen bond occurs in each anion. In the crystal, O—H⋯O and N—H⋯O hydrogen bonds link the cations and anions into a three-dimensional array. The structure is further consolidated by weak C—H⋯O inter­actions.

## Related literature

For the pharmacological activity of 3,4-dihy­droxy­benzoic acid derivatives, see: An *et al.* (2006 ▶); Lin *et al.* (2009 ▶). For related structures, see: Mazurek *et al.* (2007 ▶); Zhu (2010 ▶).
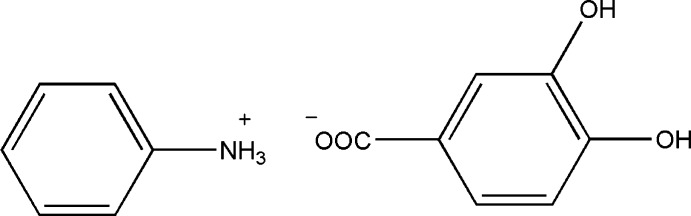


## Experimental

### 

#### Crystal data

C_6_H_8_N^+^·C_7_H_5_O_4_^−^
*M* *_r_* = 247.24
Triclinic, 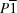

*a* = 6.8638 (16) Å
*b* = 11.566 (3) Å
*c* = 15.400 (3) Å
α = 88.980 (3)°
β = 87.517 (2)°
γ = 76.302 (2)°
*V* = 1186.6 (5) Å^3^
*Z* = 4
Mo *K*α radiation
μ = 0.10 mm^−1^
*T* = 296 K
0.30 × 0.27 × 0.27 mm

#### Data collection

Bruker APEXII area-detector diffractometer
6128 measured reflections
4202 independent reflections
3317 reflections with *I* > 2σ(*I*)
*R*_int_ = 0.024

#### Refinement

*R*[*F* ^2^ > 2σ(*F* ^2^)] = 0.039
*wR*(*F* ^2^) = 0.109
*S* = 1.05
4202 reflections
332 parameters
1 restraint
H-atom parameters constrained
Δρ_max_ = 0.18 e Å^−3^
Δρ_min_ = −0.19 e Å^−3^

### 

Data collection: *APEX2* (Bruker, 2004 ▶); cell refinement: *SAINT* (Bruker, 2004 ▶); data reduction: *SAINT*; program(s) used to solve structure: *SHELXS97* (Sheldrick, 2008 ▶); program(s) used to refine structure: *SHELXL97* (Sheldrick, 2008 ▶); molecular graphics: *SHELXTL* (Sheldrick, 2008 ▶); software used to prepare material for publication: *SHELXL97*.

## Supplementary Material

Crystal structure: contains datablock(s) I, global. DOI: 10.1107/S1600536812032758/pv2568sup1.cif

Structure factors: contains datablock(s) I. DOI: 10.1107/S1600536812032758/pv2568Isup2.hkl

Supplementary material file. DOI: 10.1107/S1600536812032758/pv2568Isup3.cml

Additional supplementary materials:  crystallographic information; 3D view; checkCIF report

## Figures and Tables

**Table 1:** Hydrogen-bond geometry (Å, °)

*D*—H⋯*A*	*D*—H	H⋯*A*	*D*⋯*A*	*D*—H⋯*A*
N1—H1*A*⋯O6^i^	0.89	1.99	2.856 (2)	165
N1—H1*B*⋯O4^ii^	0.89	2.44	2.824 (2)	107
N1—H1*B*⋯O6	0.89	2.06	2.912 (2)	161
N1—H1*C*⋯O5^iii^	0.89	1.95	2.831 (2)	170
N2—H2*A*⋯O1^iv^	0.89	2.13	2.937 (2)	150
N2—H2*A*⋯O7	0.89	2.33	2.821 (2)	115
N2—H2*B*⋯O1^v^	0.89	2.00	2.842 (2)	159
N2—H2*C*⋯O2	0.89	1.94	2.816 (2)	168
O3—H3*A*⋯O4	0.82	2.30	2.717 (2)	112
O3—H3*A*⋯O6^vi^	0.82	2.02	2.797 (2)	157
O4—H4*A*⋯O5^vii^	0.82	1.83	2.639 (2)	170
O7—H7⋯O2^v^	0.82	1.82	2.632 (2)	169
O8—H8⋯O1^iv^	0.82	2.00	2.776 (2)	158
O8—H8⋯O7	0.82	2.30	2.735 (2)	114
C25—H25⋯O3^viii^	0.93	2.53	3.385 (3)	153

Symmetry codes: (i) 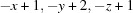; (ii) 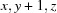; (iii) 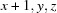; (iv) 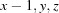; (v) 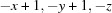; (vi) 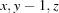; (vii) 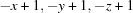; (viii) 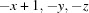.

**Fig. 1.**
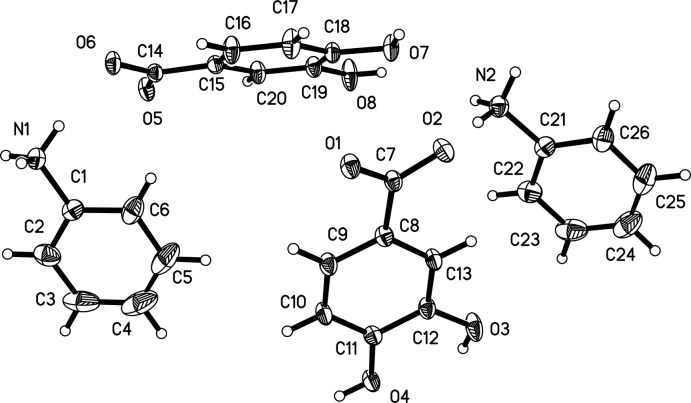
The molecular structure showing the atomic-numbering scheme and displacement ellipsoids drawn at the 30% probability level.

**Fig. 2.**
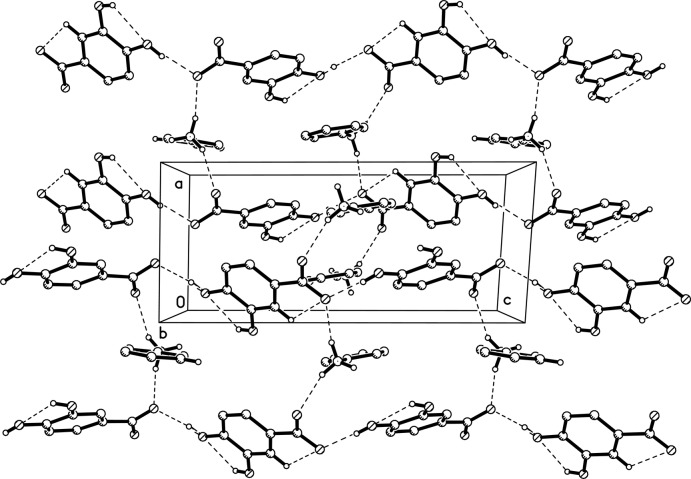
The molecular packing showing the hydrogen bonding interactions as broken lines.

## supplementary crystallographic information

### Comment

Protocatechuic acid (3,4-dihydroxybenzoic acid) and its derivatives possess diverse pharmacological activities (An *et al.*, 2006; Lin *et al.*, 2009). The molecular and crystal structure of the title compound is presented in this article.

The asymmetric unit of the title compound (Fig. 1) contains two anilinium cations and two singly deprotonated 3,4-dihydroxybenzoate anions. The bond distances and angles in the title compound agree very well with the corresponding bond distances and angles reported in closely related compounds (Mazurek *et al.*, 2007; Zhu, 2010). In the crystal the cations and anions are self-assembled by various O—H···O and N—H···O hydrogen bonds (Table 1 and Fig. 2) to form a superamolecular network. The structure is further consolidated by weak intermolecular interactions of the type C—H···O (Table 1).

### Experimental

A mixture of protocatechuic acid (0.31 g, 2 mmol) and aniline (0.19 ml, 2 mmol) was stirred in methanol (20 ml) for 0.5 h at room temperature. After several days colorless block-like crystals, suitable for X-ray diffraction analysis, were obtained by slow evaporation of the solution.

### Refinement

All H atoms were placed at calculated positions and were treated as riding, with C—H = 0.93 Å, O—H = 0.82 Å, and N—H = 0.89 Å, and *U*_iso_(H) = 1.2 or 1.5 *U*_eq_(C, O, N).

### Crystal data

**Table:**

C_6_H_8_N^+^·C_7_H_5_O_4_^−^	*Z* = 4
*M**_r_* = 247.24	*F*(000) = 520
Triclinic, *P*1	*D*_x_ = 1.384 Mg m^−^^3^
Hall symbol: -P 1	Mo *K*α radiation, λ = 0.71073 Å
*a* = 6.8638 (16) Å	Cell parameters from 2457 reflections
*b* = 11.566 (3) Å	θ = 2.2–27.3°
*c* = 15.400 (3) Å	µ = 0.10 mm^−^^1^
α = 88.980 (3)°	*T* = 296 K
β = 87.517 (2)°	Block, colorless
γ = 76.302 (2)°	0.30 × 0.27 × 0.27 mm
*V* = 1186.6 (5) Å^3^	

### Data collection

**Table:**

Bruker APEXII area-detector diffractometer	3317 reflections with *I* > 2σ(*I*)
Radiation source: fine-focus sealed tube	*R*_int_ = 0.024
Graphite monochromator	θ_max_ = 25.2°, θ_min_ = 1.8°
φ and ω scan	*h* = −8→8
6128 measured reflections	*k* = −13→11
4202 independent reflections	*l* = −18→17

### Refinement

**Table:**

Refinement on *F*^2^	Secondary atom site location: difference Fourier map
Least-squares matrix: full	Hydrogen site location: inferred from neighbouring sites
*R*[*F*^2^ > 2σ(*F*^2^)] = 0.039	H-atom parameters constrained
*wR*(*F*^2^) = 0.109	*w* = 1/[σ^2^(*F*_o_^2^) + (0.0508*P*)^2^ + 0.1856*P*] where *P* = (*F*_o_^2^ + 2*F*_c_^2^)/3
*S* = 1.05	(Δ/σ)_max_ < 0.001
4202 reflections	Δρ_max_ = 0.18 e Å^−^^3^
332 parameters	Δρ_min_ = −0.19 e Å^−^^3^
1 restraint	Extinction correction: *SHELXL97* (Sheldrick, 2008), Fc^*^=kFc[1+0.001xFc^2^λ^3^/sin(2θ)]^-1/4^
Primary atom site location: structure-invariant direct methods	Extinction coefficient: 0.077 (4)

### Special details

**Table:**

Geometry. All e.s.d.'s (except the e.s.d. in the dihedral angle between two l.s. planes) are estimated using the full covariance matrix. The cell e.s.d.'s are taken into account individually in the estimation of e.s.d.'s in distances, angles and torsion angles; correlations between e.s.d.'s in cell parameters are only used when they are defined by crystal symmetry. An approximate (isotropic) treatment of cell e.s.d.'s is used for estimating e.s.d.'s involving l.s. planes.
Refinement. Refinement of *F*^2^ against ALL reflections. The weighted *R*-factor *wR* and goodness of fit *S* are based on *F*^2^, conventional *R*-factors *R* are based on *F*, with *F* set to zero for negative *F*^2^. The threshold expression of *F*^2^ > σ(*F*^2^) is used only for calculating *R*-factors(gt) *etc*. and is not relevant to the choice of reflections for refinement. *R*-factors based on *F*^2^ are statistically about twice as large as those based on *F*, and *R*- factors based on ALL data will be even larger.

### Fractional atomic coordinates and isotropic or equivalent isotropic displacement parameters (Å^2^)

**Table:**

	*x*	*y*	*z*	*U*_iso_*/*U*_eq_	
C1	0.7332 (2)	0.75230 (15)	0.52005 (12)	0.0372 (4)	
C2	0.7644 (3)	0.71776 (19)	0.60497 (14)	0.0546 (5)	
H2	0.7840	0.7714	0.6461	0.065*	
C3	0.7662 (3)	0.6010 (3)	0.6283 (2)	0.0825 (9)	
H3	0.7869	0.5760	0.6856	0.099*	
C4	0.7375 (4)	0.5222 (2)	0.5672 (3)	0.0927 (10)	
H4	0.7389	0.4441	0.5831	0.111*	
C5	0.7070 (3)	0.5584 (2)	0.4830 (2)	0.0780 (8)	
H5	0.6887	0.5045	0.4419	0.094*	
C6	0.7028 (3)	0.67478 (17)	0.45810 (15)	0.0518 (5)	
H6	0.6801	0.6998	0.4009	0.062*	
C7	0.7273 (2)	0.39504 (14)	0.11410 (10)	0.0289 (4)	
C8	0.7123 (2)	0.32018 (14)	0.19329 (10)	0.0302 (4)	
C9	0.7779 (3)	0.34600 (15)	0.27309 (10)	0.0367 (4)	
H9	0.8273	0.4136	0.2790	0.044*	
C10	0.7701 (2)	0.27142 (15)	0.34417 (10)	0.0363 (4)	
H10	0.8132	0.2899	0.3975	0.044*	
C11	0.6993 (2)	0.17023 (14)	0.33649 (10)	0.0309 (4)	
C12	0.6287 (2)	0.14519 (14)	0.25711 (10)	0.0343 (4)	
C13	0.6371 (2)	0.21926 (14)	0.18642 (10)	0.0328 (4)	
H13	0.5917	0.2014	0.1334	0.039*	
C14	0.2555 (2)	0.87609 (14)	0.38609 (10)	0.0295 (4)	
C15	0.2338 (2)	0.80700 (14)	0.30781 (10)	0.0294 (4)	
C16	0.3636 (3)	0.79873 (16)	0.23537 (11)	0.0432 (5)	
H16	0.4660	0.8389	0.2341	0.052*	
C17	0.3413 (3)	0.73098 (17)	0.16494 (12)	0.0465 (5)	
H17	0.4290	0.7265	0.1167	0.056*	
C18	0.1917 (2)	0.67006 (14)	0.16508 (10)	0.0322 (4)	
C19	0.0601 (2)	0.67786 (14)	0.23739 (10)	0.0329 (4)	
C20	0.0831 (2)	0.74530 (15)	0.30705 (10)	0.0349 (4)	
H20	−0.0049	0.7498	0.3552	0.042*	
C21	0.2207 (2)	0.24550 (15)	0.02540 (12)	0.0368 (4)	
C22	0.1956 (3)	0.18707 (18)	0.10200 (14)	0.0498 (5)	
H22	0.1788	0.2270	0.1547	0.060*	
C23	0.1959 (3)	0.0664 (2)	0.09909 (18)	0.0691 (7)	
H23	0.1786	0.0252	0.1501	0.083*	
C24	0.2217 (3)	0.0089 (2)	0.0209 (2)	0.0729 (7)	
H24	0.2253	−0.0719	0.0193	0.087*	
C25	0.2423 (3)	0.0690 (2)	−0.05475 (18)	0.0655 (6)	
H25	0.2572	0.0292	−0.1075	0.079*	
C26	0.2411 (3)	0.18871 (17)	−0.05349 (13)	0.0474 (5)	
H26	0.2538	0.2301	−0.1049	0.057*	
O1	0.83712 (17)	0.46884 (10)	0.11452 (7)	0.0371 (3)	
O2	0.63288 (17)	0.37944 (10)	0.04814 (7)	0.0381 (3)	
O3	0.5524 (2)	0.04784 (12)	0.24703 (8)	0.0576 (4)	
H3A	0.5288	0.0209	0.2949	0.086*	
O4	0.68868 (18)	0.09263 (10)	0.40301 (7)	0.0392 (3)	
H4A	0.7455	0.1102	0.4449	0.059*	
O5	0.14211 (17)	0.87197 (11)	0.45229 (7)	0.0383 (3)	
O6	0.38799 (17)	0.93671 (10)	0.38500 (7)	0.0372 (3)	
O7	0.16366 (18)	0.60121 (11)	0.09808 (7)	0.0410 (3)	
H7	0.2382	0.6096	0.0566	0.062*	
O8	−0.0916 (2)	0.61981 (14)	0.24128 (8)	0.0564 (4)	
H8	−0.0856	0.5798	0.1974	0.085*	
N1	0.7354 (2)	0.87382 (11)	0.49340 (9)	0.0347 (3)	
H1A	0.6996	0.9221	0.5387	0.052*	
H1B	0.6496	0.8972	0.4513	0.052*	
H1C	0.8585	0.8763	0.4740	0.052*	
N2	0.2307 (2)	0.37032 (12)	0.02875 (9)	0.0370 (3)	
H2A	0.1406	0.4082	0.0682	0.055*	
H2B	0.2046	0.4042	−0.0231	0.055*	
H2C	0.3529	0.3745	0.0432	0.055*	

### Atomic displacement parameters (Å^2^)

**Table:**

	*U*^11^	*U*^22^	*U*^33^	*U*^12^	*U*^13^	*U*^23^
C1	0.0258 (8)	0.0358 (9)	0.0509 (11)	−0.0102 (7)	0.0022 (7)	0.0026 (8)
C2	0.0380 (10)	0.0650 (13)	0.0617 (13)	−0.0145 (9)	−0.0075 (9)	0.0171 (11)
C3	0.0515 (13)	0.0844 (19)	0.110 (2)	−0.0161 (13)	−0.0126 (13)	0.0570 (17)
C4	0.0596 (15)	0.0493 (15)	0.170 (3)	−0.0166 (12)	−0.0026 (18)	0.0349 (19)
C5	0.0568 (14)	0.0410 (13)	0.140 (3)	−0.0210 (11)	0.0147 (15)	−0.0136 (15)
C6	0.0416 (10)	0.0434 (11)	0.0739 (14)	−0.0179 (9)	0.0088 (10)	−0.0121 (10)
C7	0.0303 (8)	0.0346 (9)	0.0250 (8)	−0.0139 (7)	0.0005 (6)	−0.0022 (7)
C8	0.0292 (8)	0.0353 (9)	0.0290 (9)	−0.0132 (7)	0.0003 (6)	−0.0009 (7)
C9	0.0468 (10)	0.0388 (9)	0.0323 (9)	−0.0250 (8)	−0.0057 (7)	0.0009 (7)
C10	0.0451 (10)	0.0438 (10)	0.0269 (9)	−0.0229 (8)	−0.0073 (7)	−0.0020 (7)
C11	0.0337 (8)	0.0351 (9)	0.0265 (8)	−0.0140 (7)	−0.0010 (6)	0.0026 (7)
C12	0.0437 (9)	0.0360 (9)	0.0294 (9)	−0.0216 (8)	−0.0024 (7)	−0.0010 (7)
C13	0.0399 (9)	0.0392 (9)	0.0248 (8)	−0.0196 (7)	−0.0042 (7)	−0.0016 (7)
C14	0.0329 (8)	0.0316 (8)	0.0273 (8)	−0.0140 (7)	−0.0031 (7)	0.0000 (6)
C15	0.0334 (8)	0.0314 (8)	0.0258 (8)	−0.0122 (7)	−0.0023 (6)	−0.0007 (6)
C16	0.0464 (10)	0.0548 (11)	0.0395 (10)	−0.0347 (9)	0.0064 (8)	−0.0117 (8)
C17	0.0532 (11)	0.0617 (12)	0.0349 (10)	−0.0360 (10)	0.0161 (8)	−0.0164 (9)
C18	0.0410 (9)	0.0333 (9)	0.0262 (8)	−0.0164 (7)	0.0000 (7)	−0.0051 (7)
C19	0.0358 (9)	0.0391 (9)	0.0298 (9)	−0.0212 (7)	0.0009 (7)	−0.0030 (7)
C20	0.0389 (9)	0.0458 (10)	0.0258 (8)	−0.0225 (8)	0.0063 (7)	−0.0079 (7)
C21	0.0274 (8)	0.0359 (9)	0.0493 (11)	−0.0110 (7)	−0.0035 (7)	−0.0008 (8)
C22	0.0452 (11)	0.0515 (12)	0.0556 (12)	−0.0172 (9)	−0.0061 (9)	0.0093 (9)
C23	0.0564 (13)	0.0617 (15)	0.0955 (19)	−0.0266 (11)	−0.0168 (13)	0.0341 (14)
C24	0.0565 (14)	0.0427 (13)	0.123 (2)	−0.0174 (11)	−0.0112 (14)	−0.0040 (14)
C25	0.0547 (13)	0.0544 (14)	0.0902 (18)	−0.0177 (11)	0.0034 (12)	−0.0263 (13)
C26	0.0394 (10)	0.0504 (12)	0.0551 (12)	−0.0160 (9)	0.0021 (8)	−0.0113 (9)
O1	0.0453 (7)	0.0425 (7)	0.0315 (6)	−0.0267 (6)	−0.0009 (5)	0.0008 (5)
O2	0.0431 (7)	0.0504 (7)	0.0280 (6)	−0.0251 (6)	−0.0055 (5)	0.0041 (5)
O3	0.1055 (11)	0.0573 (8)	0.0308 (7)	−0.0590 (8)	−0.0125 (7)	0.0049 (6)
O4	0.0561 (8)	0.0426 (7)	0.0276 (6)	−0.0279 (6)	−0.0103 (5)	0.0056 (5)
O5	0.0409 (6)	0.0545 (8)	0.0262 (6)	−0.0248 (6)	0.0026 (5)	−0.0082 (5)
O6	0.0449 (7)	0.0436 (7)	0.0317 (6)	−0.0269 (6)	−0.0016 (5)	−0.0054 (5)
O7	0.0540 (8)	0.0497 (7)	0.0290 (6)	−0.0322 (6)	0.0094 (5)	−0.0139 (5)
O8	0.0646 (9)	0.0825 (10)	0.0408 (8)	−0.0562 (8)	0.0168 (6)	−0.0242 (7)
N1	0.0354 (7)	0.0357 (8)	0.0353 (8)	−0.0127 (6)	−0.0002 (6)	−0.0032 (6)
N2	0.0379 (8)	0.0379 (8)	0.0373 (8)	−0.0132 (6)	−0.0005 (6)	−0.0023 (6)

### Geometric parameters (Å, º)

**Table:**

C1—C2	1.371 (3)	C16—C17	1.384 (2)
C1—C6	1.376 (3)	C16—H16	0.9300
C1—N1	1.460 (2)	C17—C18	1.376 (2)
C2—C3	1.389 (3)	C17—H17	0.9300
C2—H2	0.9300	C18—O7	1.3619 (18)
C3—C4	1.374 (4)	C18—C19	1.394 (2)
C3—H3	0.9300	C19—O8	1.3646 (18)
C4—C5	1.364 (4)	C19—C20	1.374 (2)
C4—H4	0.9300	C20—H20	0.9300
C5—C6	1.387 (3)	C21—C22	1.374 (3)
C5—H5	0.9300	C21—C26	1.377 (3)
C6—H6	0.9300	C21—N2	1.463 (2)
C7—O1	1.2655 (17)	C22—C23	1.396 (3)
C7—O2	1.2663 (18)	C22—H22	0.9300
C7—C8	1.497 (2)	C23—C24	1.371 (4)
C8—C9	1.388 (2)	C23—H23	0.9300
C8—C13	1.391 (2)	C24—C25	1.364 (3)
C9—C10	1.389 (2)	C24—H24	0.9300
C9—H9	0.9300	C25—C26	1.383 (3)
C10—C11	1.378 (2)	C25—H25	0.9300
C10—H10	0.9300	C26—H26	0.9300
C11—O4	1.3609 (18)	O3—H3A	0.8200
C11—C12	1.394 (2)	O4—H4A	0.8200
C12—O3	1.3635 (18)	O7—H7	0.8200
C12—C13	1.381 (2)	O8—H8	0.8200
C13—H13	0.9300	N1—H1A	0.8900
C14—O5	1.2628 (19)	N1—H1B	0.8900
C14—O6	1.2722 (18)	N1—H1C	0.8900
C14—C15	1.490 (2)	N2—H2A	0.8900
C15—C16	1.387 (2)	N2—H2B	0.8900
C15—C20	1.389 (2)	N2—H2C	0.8900
			
C2—C1—C6	121.80 (18)	C15—C16—H16	119.8
C2—C1—N1	119.70 (17)	C18—C17—C16	121.12 (15)
C6—C1—N1	118.49 (16)	C18—C17—H17	119.4
C1—C2—C3	118.6 (2)	C16—C17—H17	119.4
C1—C2—H2	120.7	O7—C18—C17	123.75 (15)
C3—C2—H2	120.7	O7—C18—C19	117.17 (13)
C4—C3—C2	120.3 (3)	C17—C18—C19	119.08 (15)
C4—C3—H3	119.8	O8—C19—C20	118.75 (14)
C2—C3—H3	119.8	O8—C19—C18	121.81 (14)
C5—C4—C3	120.1 (2)	C20—C19—C18	119.44 (14)
C5—C4—H4	120.0	C19—C20—C15	122.05 (14)
C3—C4—H4	120.0	C19—C20—H20	119.0
C4—C5—C6	120.8 (3)	C15—C20—H20	119.0
C4—C5—H5	119.6	C22—C21—C26	121.61 (17)
C6—C5—H5	119.6	C22—C21—N2	118.65 (16)
C1—C6—C5	118.4 (2)	C26—C21—N2	119.72 (16)
C1—C6—H6	120.8	C21—C22—C23	118.6 (2)
C5—C6—H6	120.8	C21—C22—H22	120.7
O1—C7—O2	122.50 (13)	C23—C22—H22	120.7
O1—C7—C8	119.26 (13)	C24—C23—C22	119.9 (2)
O2—C7—C8	118.21 (13)	C24—C23—H23	120.1
C9—C8—C13	118.72 (14)	C22—C23—H23	120.1
C9—C8—C7	121.95 (14)	C25—C24—C23	120.7 (2)
C13—C8—C7	119.29 (14)	C25—C24—H24	119.6
C8—C9—C10	120.33 (15)	C23—C24—H24	119.6
C8—C9—H9	119.8	C24—C25—C26	120.4 (2)
C10—C9—H9	119.8	C24—C25—H25	119.8
C11—C10—C9	120.67 (15)	C26—C25—H25	119.8
C11—C10—H10	119.7	C21—C26—C25	118.8 (2)
C9—C10—H10	119.7	C21—C26—H26	120.6
O4—C11—C10	123.95 (14)	C25—C26—H26	120.6
O4—C11—C12	116.70 (13)	C12—O3—H3A	109.5
C10—C11—C12	119.33 (14)	C11—O4—H4A	109.5
O3—C12—C13	118.67 (14)	C18—O7—H7	109.5
O3—C12—C11	121.43 (14)	C19—O8—H8	109.5
C13—C12—C11	119.90 (14)	C1—N1—H1A	109.5
C12—C13—C8	121.02 (14)	C1—N1—H1B	109.5
C12—C13—H13	119.5	H1A—N1—H1B	109.5
C8—C13—H13	119.5	C1—N1—H1C	109.5
O5—C14—O6	121.70 (14)	H1A—N1—H1C	109.5
O5—C14—C15	118.76 (13)	H1B—N1—H1C	109.5
O6—C14—C15	119.54 (13)	C21—N2—H2A	109.5
C16—C15—C20	117.95 (14)	C21—N2—H2B	109.5
C16—C15—C14	122.22 (14)	H2A—N2—H2B	109.5
C20—C15—C14	119.81 (14)	C21—N2—H2C	109.5
C17—C16—C15	120.36 (15)	H2A—N2—H2C	109.5
C17—C16—H16	119.8	H2B—N2—H2C	109.5

### Hydrogen-bond geometry (Å, º)

**Table:**

*D*—H···*A*	*D*—H	H···*A*	*D*···*A*	*D*—H···*A*
N1—H1*A*···O6^i^	0.89	1.99	2.856 (2)	165
N1—H1*B*···O4^ii^	0.89	2.44	2.824 (2)	107
N1—H1*B*···O6	0.89	2.06	2.912 (2)	161
N1—H1*C*···O5^iii^	0.89	1.95	2.831 (2)	170
N2—H2*A*···O1^iv^	0.89	2.13	2.937 (2)	150
N2—H2*A*···O7	0.89	2.33	2.821 (2)	115
N2—H2*B*···O1^v^	0.89	2.00	2.842 (2)	159
N2—H2*C*···O2	0.89	1.94	2.816 (2)	168
O3—H3*A*···O4	0.82	2.30	2.717 (2)	112
O3—H3*A*···O6^vi^	0.82	2.02	2.797 (2)	157
O4—H4*A*···O5^vii^	0.82	1.83	2.639 (2)	170
O7—H7···O2^v^	0.82	1.82	2.632 (2)	169
O8—H8···O1^iv^	0.82	2.00	2.776 (2)	158
O8—H8···O7	0.82	2.30	2.735 (2)	114
C20—H20···O5	0.93	2.48	2.791 (2)	100
C25—H25···O3^viii^	0.93	2.53	3.385 (3)	153

Symmetry codes: (i) −*x*+1, −*y*+2, −*z*+1; (ii) *x*, *y*+1, *z*; (iii) *x*+1, *y*, *z*; (iv) *x*−1, *y*, *z*; (v) −*x*+1, −*y*+1, −*z*; (vi) *x*, *y*−1, *z*; (vii) −*x*+1, −*y*+1, −*z*+1; (viii) −*x*+1, −*y*, −*z*.
